# Case report: Focal segmental glomerulosclerosis and nephrotic syndrome following treatment with pamidronate for calcitriol toxicity

**DOI:** 10.3389/fvets.2022.956153

**Published:** 2022-08-12

**Authors:** Katherine Ann Dawson, April Blong, Rebecca Walton

**Affiliations:** Department of Veterinary Clinical Sciences, College of Veterinary Medicine, Iowa State University, Ames, IA, United States

**Keywords:** hypercalcemia, pamidronate, protein losing nephropathy, calcitriol (1, 25(OH) 2D 3), toxicity

## Abstract

**Objective:**

This study aimed to describe a case of glomerulosclerosis resulting in nephrotic syndrome following the administration of pamidronate disodium to treat clinical calcitriol toxicity in a dog.

**Case summary:**

A 12-week-old intact male Labrador Retriever weighing 11.8 kg presented with lethargy and vomiting for 20 h after ingesting a 100 g tube of topical antipsoriatic cream (3 mcg/g of calcitriol; Vectical Ointment™, Galderma, Lausanne, Switzerland). Severe hypercalcemia was present on the day of the presentation. Hypercalcemia treatments such as saline diuresis, furosemide (Salix^®^, furosemide, Merck Animal Health, Kenilworth, NJ), and dexamethasone sodium phosphate (Dexamethasone SP, Mylan, Canonsburg, PA) were initiated. The dog was also administered a single dose of pamidronate disodium (Pamidronate disodium, Mylan, Canonsburg, PA) on the day of presentation. Initially, the patient's clinical signs improved, and the hypercalcemia resolved. Exactly 130 h post-pamidronate disodium (Dexamethasone SP, Mylan, Canonsburg, PA) administration, the patient developed biochemical abnormalities and severe edema, consistent with nephrotic syndrome, and was euthanized. Necropsy results revealed evidence of focal segmental glomerulosclerosis (FSGS).

**Unique information:**

Pamidronate disodium, commonly used for the treatment of hypercalcemia, may have resulted in glomerulosclerosis and nephrotic syndrome in a dog with calcitriol toxicity. This complication should be taken into consideration when monitoring patients treated with pamidronate disodium for hypercalcemia.

## Introduction

Calcitriol, the most biologically active form of vitamin D, is commonly used to treat psoriasis in people. Accidental ingestion in dogs can result in life-threatening hypercalcemia (1, 2). In the therapeutic setting, calcitriol's mechanism of action in treating psoriasis is *via* inhibition of keratinocyte proliferation in combination with an immunomodulatory role through the suppression of pro-inflammatory cytokines and the stimulation of anti-inflammatory cytokines (3, 4). With acute intoxication, calcitriol increases calcium absorption from the gastrointestinal tract, stimulates bone resorption, and increases calcium absorption in the renal distal tubules (5). The combination of these effects results in increased serum calcium and phosphorus, resulting in soft tissue mineralization (5).

Many reports in veterinary medicine describe the routine and successful use of bisphosphonates, such as second-generation bisphosphate, and pamidronate, in the management of hypercalcemia for a variety of etiologies (6–10). The only documented complications associated with pamidronate administration in veterinary medicine include mild electrolyte disturbances and extravasation (11, 12). Bisphosphonates, such as pamidronate, decrease serum ionized calcium by inhibiting osteoclastic activity and induction of osteoclast apoptosis (13). Despite the reported safe and routine use of pamidronate in dogs, several cases reports in human medicine describe glomerulosclerosis and resulting nephrotic syndrome in patients that received intravenous pamidronate (14–17). Acute kidney injury and nephrotoxicity are mentioned complications of pamidronate in dogs; however, this risk has only been extrapolated from human medicine with no documented cases in dogs (6, 9). A third-generation bisphosphate, zoledronic acid, has been documented to result in a mild acute kidney injury in cancer-bearing dogs, however, nephrotic syndrome has never been previously reported in veterinary medicine (18). The purpose of this report is to describe a never-reported potential complication of pamidronate administration in a dog for treatment of calcitriol toxicity and hypercalcemia.

## Case summary

A 12-week-old intact male Labrador Retriever dog weighing 11.8 kg (25.96 lb) presented to the Emergency Department of a University Teaching Hospital for the evaluation and treatment of hypercalcemia following ingestion of a total of 300 mcg of calcitriol in the form of an anti-psoriasis cream (Vectical Ointment™, Galderma, Lausanne, Switzerland). The dog ingested the medication 20-h prior to presentation at an estimated dose of 25 mcg/kg of calcitriol. Vomiting and progressive lethargy were noted within 12 h of ingestion. The dog was initially evaluated by the primary care provider for 18 h (day one) after ingestion of the toxicant. A total serum calcium concentration of >16 mg/dl (reference range 7.8–12.6 mg/dl) was noted on the serum biochemical profile (Table 1). After the discovery of the severe total hypercalcemia, the dog was referred for further management of severe hypercalcemia about 20 h post-ingestion.

**Table 1:** Summary of pertinent serum chemistry findings.

	**Day 1 (referring)**	**Day 1 (intake)**	**Day 2**	**Day 3**	**Day 4**	**Day 6**	**Reference interval**
Glucose mmol/L (mg/dl)	6.94 (125)	6.16 (111)	6.88 (124)	NE	NE	6.72 (121)	3.77–6.38 (68–115)
Creatinine μmol/L (mg/dl)	53.05 (0.6)	88.42 (1)	132.63 (1.5)	123.79 (1.4)	97.26 (1.1)	168 (1.9)	44.21–123.79 (0.5–1.4)
BUN mmol/L (mg/dl)	8.47 (24)	8.57 (36)	16.07 (45)	13.21 (37)	14.28 (40)	17.5 (49)	3.57–10.71 (10–30)
Phosphorous mmol/L (mg/dl)	3.84 (11.9)	4.75 (14.7)	3 (9.3)	2.84 (8.8)	2.39 (7.4)	2.39 (7.4)	1.03–1.94 (3.2–6.0)
Total protein g/dl	5.7	4.8	3.2	NE	NE	3.5	5.2–7.1
Albumin (g/dl)	2.8	3.3	1.5	1.2	1.2	1.6	2.7–4.0
Cholesterol mmol/L (mg/dl)	5.96 (230)	NE	6.99 (270)	NE	NE	9.87 (381)	3.42–7.77 (132–300)
Ionized calcium mmol/L	NE	1.77	1.72	1.81	1.37	1.13	1.25–1.45

NE, not examined.

The patient's vitals on presentation were 39.5°C (103.1°F), 210 beats per min, and 72 breaths per min. Physical examination upon presentation (day one) was consistent with 5% dehydration, moderate abdominal pain on palpation, harsh lung sounds ventrally, and quiet mentation. On venous blood gas, ionized hypercalcemia at 1.72 mmol/L (reference range 1.25–1.45 mmol/L) was present. Serum biochemical abnormalities included total hypercalcemia [>4 mmol/L, reference range 2.42–2.78 mmol/L (> 16 mg/dl reference range 9.7–11.13 mg/dl)] and hyperphosphatemia [4.75 mmol/L; reference range 1.03–1.94 mmol/L (14.7 mg/dl; reference range 3.2–6 mg/dl); Table 1]. At this time, albumin 3.3 gm/dl (reference range 2.5–4.4 mg/dl) and creatinine 88.42 μmol/L [44.21 μmol/L-132.63 μmol/L (1 mg/dl reference range (reference range. 5–1.5 mg/dl))] were both within reference interval (Table 1). Survey radiographs were consistent with a mild, ventral mixed pulmonary pattern attributed to suspected early aspiration pneumonia given the history of vomiting. There was additional evidence of bilateral renomegaly, attributed to potential acute kidney injury. Initial therapeutic interventions consisted of therapies to decrease serum calcium and preserve kidney function. A balanced electrolyte solution (Normosol-R^®^, Icu-Medical, Inc., Lake Forest, IL) was started intravenously at a rate of 183 ml/kg/day, in addition to ampicillin/sulbactam (Unasyn^®^, Pfizer, New York, NY; 30 mg/kg IV q 8 h), fentanyl (Fentanyl, Hospira, Lake Forrest, IL; 3 mcg/kg/h) for the observed abdominal pain, maropitant (Cerenia^®^, Pfizer, New York, NY; 1 mg/kg IV q 24 h) and unilateral nasal oxygen insufflation at a rate of 200 ml/kg/min due to suspected aspiration pneumonia and perceived increase in respiratory effort. The patient was decontaminated with cholestyramine (Cholestyramine, Sandoz, Basel, Switzerland; 0.68 gm/kg PO q 8 h, eight total doses). A single dose of pamidronate disodium (Dexamethasone SP, Mylan, Canonsburg, PA) was administered within 3 h of admission (day one). A 2 mg/kg dose of pamidronate disodium (Dexamethasone SP, Mylan, Canonsburg, PA) was diluted into 250 ml of. 9% sodium chloride (0.9% Sodium Chloride, Braun Medical Inc., Ontario, Canada) and was administered intravenously over 2 h. Later that evening, the dog developed facial edema, which was attributed to being an acute type I hypersensitivity reaction post ampicillin/sulbactam (Unasyn^®^, Pfizer, New York, NY) administration. The patient was given diphenhydramine (Diphenhydramine, West-Ward Pharmaceuticals, London, England; 2 mg/kg IM) and the facial swelling rapidly resolved. By the evening of the presentation, the dog's somnolence improved, and he was observed to be readily eating.

On day 2 of hospitalization, the dog's clinical status improved, however, the hypercalcemia was static at ionized calcium of 1.72 mmol/L (reference range 1.25–1.45 mmol/L, Table 1). The dog's weight had increased to 13 kg, which was assessed to be the dog's euhydrated weight. Due to the persistent hypercalcemia, therapies were altered to increase calciuresis. The dog was switched to 0.9% sodium chloride (0.9% Sodium Chloride, Braun Medical Inc., Ontario, Canada; 150 ml/kg/day), administered dexamethasone sodium phosphate (Dexamethasone SP, Mylan, Canonsburg, PA; 0.15 mg/kg IV q 8 h), and furosemide (Salix^®^, furosemide, Merck Animal Health, Kenilworth, NJ; 5 mg/kg IV q 8 h). The dog's physical exam improved with continued euhydration, improved lung sounds, and less pain noted on abdominal palpation. The patient's pulse oximetry was persistently 98%−100% on 200 ml/kg/min of nasal oxygen, which was decreased to 100 ml/kg/min due to improved lung sounds, oxygenation, and respiratory effort. The patient's biochemistry values worsened as there was a newly developed acute kidney injury, hyperphosphatemia, and panhypoproteinemia (Table 1). Given the degree of hyperphosphatemia, aluminum hydroxide (Aluminum hydroxide, Rugby Laboratories, Livonia, MI; 150 mg/kg/day divided PO) was instituted.

On days 3 and 4 of hospitalization, the patient continued to improve. The dog was weaned off oxygen and analgesia and was reliably eating resting energy requirement by mouth. The patient's pulse oximetry was persistently 95% on room air. The dog's weight was static at 13.4 kg and the ionized calcium improved to 1.25 mmol/L (reference range 1.25–1.45 mmol/L) as did the patient's acute kidney injury and hyperphosphatemia (Table 1). Given the improved calcium, intravenous fluid therapy was transitioned back to a balanced electrolyte solution (Normosol-R^®^, Icu-Medical, Inc., Lake Forest, IL; 150 ml/kg/day). Despite the improved calcium, the dog's hypoalbuminemia worsened (Table 1).

On day 5, the patient was assessed to be slightly more quiet than the previous and was not eating by mouth. Peripheral edema was noted on all four legs, the face, and the ventral abdomen that was progressive throughout the day. The patient has slowly tapered off fluids which were discontinued at 8:00 PM on day 5 as the patient's weight had increased to 14.2 kg, at that time the ionized calcium was normal at 1.25 mmol/L (reference range 1.25–1.45 mmol/L; Table 1)

By day 6 of hospitalization, the patient was markedly lethargic, and had developed marked, diffuse peripheral edema, and abdominal distention with an expiratory push on respiration noted. On point of care ultrasound, the dog had developed both pleural and peritoneal effusion. A diagnostic thoracocentesis and abdominocentesis confirmed the cavitary effusions were both pure transudates. On laboratory evaluation, the patient had developed ionized hypocalcemia at 1.13 mmol/L (reference range 1.25–1.45 mmol/L), worsened azotemia, and improved hypoalbuminemia; both findings suspected a large degree of third-spacing of fluid. New hypercholesterolemia was also noted (Table 1). A urinalysis was performed and marked proteinuria was noted with an increased urine protein creatinine ratio of 25 (reference range 0–1). Due to the rapidly declining clinical status, poor prognosis, and exhausted owner finances, the dog was humanely euthanized 6 days after hospitalization and about 130 h after pamidronate disodium administration.

On post-mortem evaluation, the glomeruli were segmental to globally hypercellular with occasional effacement of capillary tufts, consistent with focal segmental glomerulosclerosis (FSGS). No soft tissue mineralization of the kidneys or other soft tissue was noted.

## Discussion

Calcitriol is the most metabolically active form of Vitamin D, therapeutically, it is utilized for a variety of conditions in both human and veterinary medicine and is the active ingredient in many psoriasis creams (1, 4). Ingestion of calcitriol leads to hypercalcemia *via* increasing intestinal absorption of calcium, the release of calcium stores from bone, and increased absorption of calcium in the kidney (5, 19). Hypercalcemia typically occurs within 24 h of ingestion and the onset of clinical signs of calcitriol toxicity usually occurs within 12–36 h of ingestion and includes: vomiting, anorexia, diarrhea, polyuria, polydipsia, depression, and weakness (1, 5, 19). Additionally, serum phosphorus concentrations increase through increased resorption from bone, increased renal tubular re-absorption, and increased intestinal absorption (1, 5, 19). As a result of increasing calcium-phosphorus-product, soft tissue mineralization of the heart, gastrointestinal tract, lungs, and kidneys occurs, contributing to the potentially lethal side effects (1, 5, 19). Within the kidney, vasoconstriction of the afferent arteriole, mineralization of the tubular basement membrane, and tubular degeneration result in profound azotemia and kidney failure if left untreated (5, 19).

Traditionally, treatment of calcitriol toxicosis is aimed at decreasing calcium reabsorption, promoting calcium excretion, and reducing osteoclast activity (5). Mainstays include saline diuresis, furosemide, and corticosteroid administration (5, 7, 19). Several case reports also report positive outcomes in dogs treated with bisphosphonates (6–10). Bisphosphonates aid in the treatment of hypercalcemia secondary to calcitriol toxicosis by reducing osteoclast activity and stimulating osteoclast apoptosis (7, 9, 13). In people following intravenous administration of pamidronate disodium, ~50% of the drug is incorporated into the bone to reduce osteoclast activity while the remainder of pamidronate is excreted unchanged in the urine (13, 20). Although the benefits of pamidronate disodium are well documented, several reports demonstrate both acute tubular necrosis and glomerulopathies in humans associated with its administration (8, 14–17). In people, rapid administration (within <5 min) and high doses of pamidronate disodium (>2–4 times the standard 60 mg per person dose) resulted in acute kidney injury in patients with mammary and prostatic neoplasia (15, 17, 20). Other reports have repeatedly demonstrated the histopathologic finding of FSGS and resulting nephrotic syndrome after usage of pamidronate in oncologic patients, including a case report of FSGS after dose escalation in a patient with no pre-existing kidney insufficiency (16, 17). Additionally, two patients were reported to develop nephrotic syndrome and progressive loss of renal function following treatment with pamidronate (17). Renal biopsies in these cases revealed collapsing FSGS (17). Overall risk factors for the development of nephrotoxicity or glomerulopathy in people seem to be dose-dependent, infusion-length-dependent, and potentially related to preexisting kidney insufficiency. It is worth noting many of these patients were also undergoing various chemotherapies (14–17).

In veterinary patients, few side effects are reported after usage of pamidronate disodium. A case series in 11 dogs reported mild to moderate tissue necrosis after pamidronate extravasation reactions (11). A second case reports arrhythmias after pamidronate extravasation, that were suspected to be secondary to hypomagnesemia rather than the direct cardiotoxicity of pamidronate disodium (12). In the patient in this report, FSGS was identified on histopathology of the kidneys, which is the characteristic histopathologic diagnosis in people that develop nephrotic syndrome after pamidronate therapy (14–17). The postulated mechanism of nephrotoxicity involves similar cellular effects as to how bisphosphonates inhibit osteoclasts (13, 16). When bisphosphonates are internalized by osteoclasts, cellular activity is inhibited, and apoptosis occurs by the inhibition of enzymes responsible for the production of cholesterol and lipids. Bisphosphonates also disrupt guanosine triphosphate transmembrane proteins which in turn halt vital cell signaling pathways (13, 16). It is theorized that similar changes occur within the glomerular podocytes, which may play a role in the development of glomerulosclerosis. Additionally, both osteoclasts and podocytes possess a similarly unique cytoskeleton and bisphosphonates inhibit actin rings which can disrupt the cytoskeleton of both osteoclasts and podocytes resulting in apoptosis (16).

In this patient, FSGS occurred after a single 2 mg/kg dose of pamidronate was infused over 2 h. This dosage was on the high-end of the effective doses of pamidronate studied in dogs and was not considered to be a rapid infusion (7). Establishing causality between a therapeutic agent and disease process can be difficult, especially in a single case report. Additionally, the hypercalcemia documented in this case may have contributed to the renal dysfunction and resulting nephrotic syndrome, however, it was deemed less likely due to the histopathologic changes to the glomeruli noted and the lack of any soft tissue mineralization appreciated on necropsy. Overall, the development of severe protein-losing nephropathy and nephrotic syndrome in the case may be multi-factorial, however, due to the similarities noted between this case and people with pamidronate administration this report may serve as a caution when considering pamidronate usage.

In conclusion, pamidronate is a widely used and important therapeutic agent in the treatment of toxicity-induced hypercalcemia and in oncologic patients. This case report serves to describe a potential complication of pamidronate administration in the form of FSGS and nephrotic syndrome and we recommend potential periodic assessment for proteinuria and renal insufficiency in patients receiving this treatment.

## Data availability statement

The original contributions presented in the study are included in the article/supplementary material, further inquiries can be directed to the corresponding author.

## Author contributions

KD, AB, and RW contributed to manuscript writing and all edits. All authors contributed to the article and approved the submitted version.

## Conflict of interest

The authors declare that the research was conducted in the absence of any commercial or financial relationships that could be construed as a potential conflict of interest.

## Publisher's note

All claims expressed in this article are solely those of the authors and do not necessarily represent those of their affiliated organizations, or those of the publisher, the editors and the reviewers. Any product that may be evaluated in this article, or claim that may be made by its manufacturer, is not guaranteed or endorsed by the publisher.

## Abbreviations

FSGS: focal segmental glomerulosclerosis.
